# Resilience in 2021—Descriptive Analysis of Individuals Accessing Virtual Mental Health Services: Retrospective Observational Study

**DOI:** 10.2196/34283

**Published:** 2022-03-31

**Authors:** Grant Graziani, Sarah Kunkle, Emily Shih

**Affiliations:** 1 Ginger San Francisco, CA United States

**Keywords:** mental health, resilience, adaptability, measures, digital health, virtual health, psychiatry, demographic, depression, anxiety, symptom, support, treatment

## Abstract

**Background:**

Psychological resilience has been extensively studied by developmental researchers, and there is a growing body of literature regarding its role in psychiatry and psychopathology research and practice. This study contributes to this growing literature by providing real-world evidence on the relationship between resilience and clinical symptoms among a large sample of employed Americans.

**Objective:**

This study aimed to describe resilience levels in individuals accessing Ginger, a virtual mental health system, in addition to the association of resilience with demographic characteristics, baseline depression, and anxiety symptoms.

**Methods:**

We conducted a retrospective observational study of 9165 members who signed up for Ginger and completed a baseline survey between January 1 and August 5, 2021. We used multivariate regression models to test for associations between baseline resilience and other member characteristics.

**Results:**

Baseline resilience scores centered on a mean of 23.84 (SD 6.56) and median of 24 (IQR 8) out of 40, with 81.0% (7424/9165) of the sample having low resilience at baseline. Despite having relatively higher resilience scores, members with no or mild depression or anxiety still had low resilience scores on average. Self-reported suicidal ideation was associated with lower resilience.

**Conclusions:**

Overall, members had low baseline resilience, similar to resilience levels observed in trauma survivors in prior studies. Younger members and those with higher levels of depression and anxiety at intake reported lower levels of resilience at baseline. Notably, members with no or mild depression or anxiety still had low resilience scores on average, suggesting a need for mental health support among individuals who might not typically be recommended for treatment based on traditional clinical assessments, such as the 9-item Patient Health Questionnaire (PHQ-9) and the 7-item Generalized Anxiety Disorder scale (GAD-7). Two suggestions for topics of future research are to develop treatment recommendations based on the Connor-Davidson Resilience Scale and to understand the interaction between resilience levels and symptom-based outcome measures, such as the PHQ-9 and the GAD-7.

## Introduction

### Resilience and Adaptability

Psychological resilience, subsequently referred to as resilience, represents the personal qualities that enable an individual to thrive in the face of adversity. It can be viewed as a measure of the ability to cope with stress and is potentially an important target of treatment for anxiety, depression, and stress reactions, such as posttraumatic stress disorder (PTSD). Research has shown that resilience is a multidimensional characteristic and that it may vary with context, time, age, gender, and cultural origin, as well as different life circumstances [1,2].

The construct of resilience has long been of interest to developmental psychologists [3]. Considered a personal strength, resilience can contribute to positive functioning and optimal development and can prevent negative emotions, thoughts, and behaviors [4]. While it does not imply total invulnerability to the development of psychiatric disorders, resilience serves as a protective factor against the development and onset of psychopathology [3]. An evaluation of hypotheses about the relationship of resilience to personality traits, coping, and psychiatric symptoms found that it demonstrates strong positive correlations with extraversion and conscientiousness, and it can moderate the relationship between retrospective reports of childhood emotional neglect and current psychiatric symptoms [3]. Researchers have also pointed out that resilience is not necessarily a feature of an individual’s internal psychological processes but can be a product of an individual’s social and psychological ecosystem, including individual, family, community, and cultural factors [5].

There is a growing body of literature on resilience in the fields of psychiatry and psychopathology, which tends to be more focused on disease and pathology. A new approach in psychopathology research, advocated by some authors, focuses on positive adaptation in response to stress [3]. As part of this approach, there is interest in moving beyond an emphasis on pathology and focusing on prevention through human strengths and protective factors [6]. The importance of resilience as an inherent ability to manage daily stresses, as well as to overcome severe trauma, has increasingly been recognized [7]. Studies of frontline health care workers during the beginning of the COVID-19 pandemic and responders to the September 11, 2001, attacks have shown that resilience is important for both risk of PTSD and the ability to overcome trauma [8,9]. Further, researchers have made progress modeling the neurobiological components of resilience, pointing to both physiological processes and genetic factors that shape a person’s resilience [10].

### COVID-19

The COVID-19 pandemic presented the need for a prolonged period of social distancing and potential isolation, a changing and uncertain time frame for improvement in conditions and lifting of restrictions, and uncertain political and economic implications. Recent studies have shown that individuals with lower resilience scores experienced increased odds of mental distress and expressed greater difficulty coping with the emotional challenges of the pandemic crisis [11,12]. Given these unique challenges to resilience and mental well-being, more attention is being given to increasing resilience from both research and clinical perspectives. In particular, health systems have recognized the need to promote resilience among both health care workers and patients. As New York City became the epicenter of the pandemic in the United States, the Mount Sinai Health System created the Center for Stress, Resilience, and Personal Growth in order to address the pandemic’s psychological impact on the health care workers in the system; they also created a resilience app, the Wellness Hub, as a standalone digital platform offering users a suite of tools that they could interact with on a daily basis [13]. Furthermore, the pandemic has spurred increased interest in not only understanding the neurobiological and cultural process that shape resilience but also in developing interventions that improve resilience, as individuals around the globe cope with the implications of the COVID-19 pandemic [14]. In this regard, digital health platforms can play an important role in delivering such interventions.

### Study Objectives

Given current events, increased demand for mental health services, and newer disciplines like behavioral health coaching, it is important to understand individual needs, particularly those that might not be captured in traditional clinical assessments, such as the 9-item Patient Health Questionnaire (PHQ-9) and the 7-item Generalized Anxiety Disorder scale (GAD-7). With this in mind, Ginger, an on-demand mental health system, began collecting self-reported resilience data from its members beginning in December 2020. These data are used in this study, which aims to describe resilience levels in individuals accessing virtual mental health services, a population that is less understood given the relatively nascent industry. In particular, we aim to answer the following research questions: (1) What is the distribution of baseline resilience? and (2) To what extent are baseline scores correlated with demographic characteristics and concurrent depression and anxiety symptoms?

## Methods

### Overview

This is a retrospective observational study of individuals who accessed Ginger. Data were collected from Ginger members between January 1 and August 5, 2021.

### The Ginger System

Ginger provides virtual on-demand mental health services, primarily through employee or health plan benefits. Via a mobile app platform, Ginger members can access behavioral health coaching, teletherapy, and telepsychiatry, as well as self-guided content and assessments. This system has been described in more detail in prior publications evaluating depression and anxiety outcomes as measured by the PHQ-9 and the GAD-7 [15,16].

### Participants

Study participants had access to the Ginger system as part of their employer or health plan benefits. Internal clinical protocols include the following exclusionary criteria where self-directed telehealth is likely not appropriate and where more specialized and urgent psychiatric services are required:

1. Active suicidal ideation.
2. Active high-risk self-harm behavior.
3. Two or more hospitalizations within the past 6 months or one hospitalization in the past month for psychiatric reasons.
4. Certain symptoms of psychosis that are poorly managed (eg, member is not medication-compliant or symptoms are unresponsive to treatment) and are likely incompatible with telehealth.
5. A primary diagnosis of a substance use disorder or moderate to severe substance abuse issues, due to the high complexity, severity, and risk frequently associated with such members, as well as the need for specialized care.
6. Active eating disorders with symptoms considered to be high risk.
7. Ongoing grave disability, including certain patients who are bipolar with active mania, hypomania, or mixed episodes; are unmedicated; or have poor compliance with a medication regimen over time.
8. Two or more medical hospitalizations in the last month, due to the high likelihood that the individual has a poorly controlled medical condition that requires close monitoring.

For this study, we included Ginger users aged 18 years or older who joined during the study data collection period.

### Data Collection

**Figure 1:**
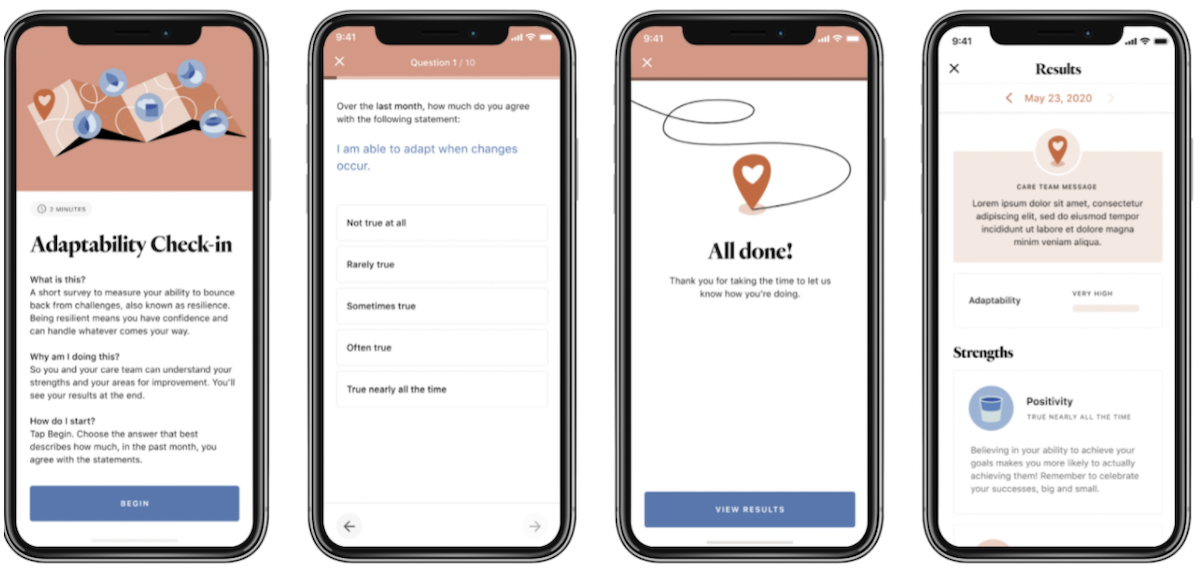
Screenshots of the Ginger mobile app showing the 10-item Connor-Davidson Resilience Scale.

As part of its measurement-based care system, Ginger uses various assessments, including the PHQ-9 and the GAD-7. Since December 2020, Ginger has used the 10-item Connor-Davidson Resilience Scale (CD-RISC 10), also referred to as an adaptability check-in, to track progress beyond depression and anxiety symptoms. This is particularly relevant to understand needs of “subclinical” members (ie, those members who screen negative for depression or anxiety at intake). This measure was selected due to behavioral health coaching’s focus on building resilience and its strength-based focus in contrast to more traditional symptom measures, such as the PHQ-9 and the GAD-7. The CD-RISC 10 was sent to members 1 week after they signed up, and a follow-up survey was sent to members every 30 days. Importantly, members who signed up but did not engage with the app past the 1-week mark did not complete the baseline survey. In this way, members with a low likelihood of meaningful engagement, which is a proxy for behavioral health need, were excluded from the sample. Visuals of how this survey appeared to members are shown in Figure 1.

### Measures

#### The Connor-Davidson Resilience Scale

As mentioned above, Ginger uses the CD-RISC 10 to measure self-reported perceived resilience. The development of the Connor-Davidson Resilience Scale (CD-RISC) [1] arose from the researchers’ extensive treatment of individuals suffering from PTSD. They initially developed a 25-item scale to measure resilience, or how well one is able to adapt to change and bounce back after stressful events, tragedy, or trauma. Two briefer versions, the CD-RISC 10 [17] and the 2-item CD-RISC [18], were subsequently developed by other research teams. The CD-RISC 10 has demonstrated robust validity, reliability, and practicality [1]. Since its development in 2003, the CD-RISC has been translated into many different languages and studied in a variety of populations [1].

The CD-RISC 10 contains 10 of the original 25 items from the CD-RISC. The 10 topics included in the CD-RISC 10 are as follows: confidence, determination, flexibility, focus, grit, perseverance, personal growth, positivity, self-reliance, and weathering emotions. For each of the 10 items, respondents were asked to select one of the following responses to a statement (eg, “I am able to adapt when changes occur”): not true at all (0), rarely true (1), sometimes true (2), often true (3), and true nearly all the time (4). A respondent’s total score could range from 0 to 40. Results from the US population indicated that the quartiles for this measure are as follows: quartile 1, 0 to 29 points; quartile 2, 30 to 32 points; quartile 3, 33 to 36 points; and quartile 4, 37 to 40 points [17].

#### Baseline Characteristics

For each member, the following data were either collected at baseline or were fixed characteristics of members: age group, gender, geographic region, PHQ-9 score, and GAD-7 score. The demographic and location data were not self-reported. Instead, they were reported by a member’s parent organization, which was either their employer or their health insurance plan. The baseline PHQ-9 and GAD-7 data were collected within the Ginger system. Baseline PHQ-9 and GAD-7 scores were selected by looking at the window from 1 week before to 1 week after a member’s baseline CD-RISC 10 score was collected and choosing the first PHQ-9 and GAD-7 scores in that window.

For many Ginger members in this study, baseline characteristics were missing. Data were missing due to one of two reasons. First, a member’s parent organization may not have shared the member’s demographic information. Thus, missing demographic data is a signal of a member’s parent organization and not necessarily a signal of information specific to a given member. For example, of the 249 parent organizations represented in this study, 116 (46.6%) reported all of their members’ gender information and 121 (48.6%) did not report their members’ gender information. The remaining 12 (4.8%) reported gender information for some but not all of their members. Second, there may not have been a PHQ-9 or GAD-7 score within the 1-week window around the collection of a member’s baseline CD-RISC 10 score. This could be due to a member not completing the PHQ-9 or the GAD-7 at all or due to the timing of their completion of surveys falling outside the 2-week window.

### Analyses

#### Sample

This study included 9165 Ginger members who completed a baseline survey at any point from January 1 to August 5, 2021. This sample will be referred to as the baseline sample.

#### Summary Statistics and Subgroup Analysis

Our descriptive analysis summarized baseline resilience scores and presented the mean (SD) and median (IQR) of baseline scores. We used a Welch *t* test to analyze differences in means across subgroups of members with unequal variances when a category had two groups (eg, gender). For categories with more than two groups (eg, census regions), we used an *F* test as part of an analysis of variance to test for significant differences in means across the groups. Further, to understand whether members with missing data had significantly different outcomes than those without missing data, we performed Welch 2-tailed *t* tests comparing means across the two groups.

#### Descriptive Multivariate Regressions

In order to understand the associations between resilience and specific covariates, we leveraged a multivariate regression model as part of our descriptive analysis of baseline scores. This methodology accounted for possible correlations among covariates and isolated the relationship between each covariate and resilience, holding all other covariates constant. We estimated an ordinary least squares (OLS) linear regression predicting baseline scores. The following categorical independent variables were included in the model: gender, age group, census region, and indicators for whether a member’s baseline PHQ-9 and GAD-7 scores were each above 10. For each of these independent variables, a category for members with missing data was included. No interaction terms were included, although, theoretically, there could be significant differences at a more granular level. Additionally, self-reported suicidal ideation (ie, indicating more frequently than “never” on question 9 of the PHQ-9) was included as an indicator variable. Homoscedasticity was not assumed, and robust standard errors were computed.

### Ethical Considerations

This study represents a secondary analysis of pre-existing deidentified data. The study team does not have access to the participants or to the participants’ identifying information and does not intend to recontact participants. This study protocol was reviewed by the Advarra Institutional Review Board (IRB) and determined to be exempt from IRB oversight, as deidentified secondary data analysis is generally not regarded as human subjects research; this is in accordance with the US Department of Health and Human Services regulations for the protection of human subjects in research (45 CFR 46) [19].

## Results

**Figure 2:**
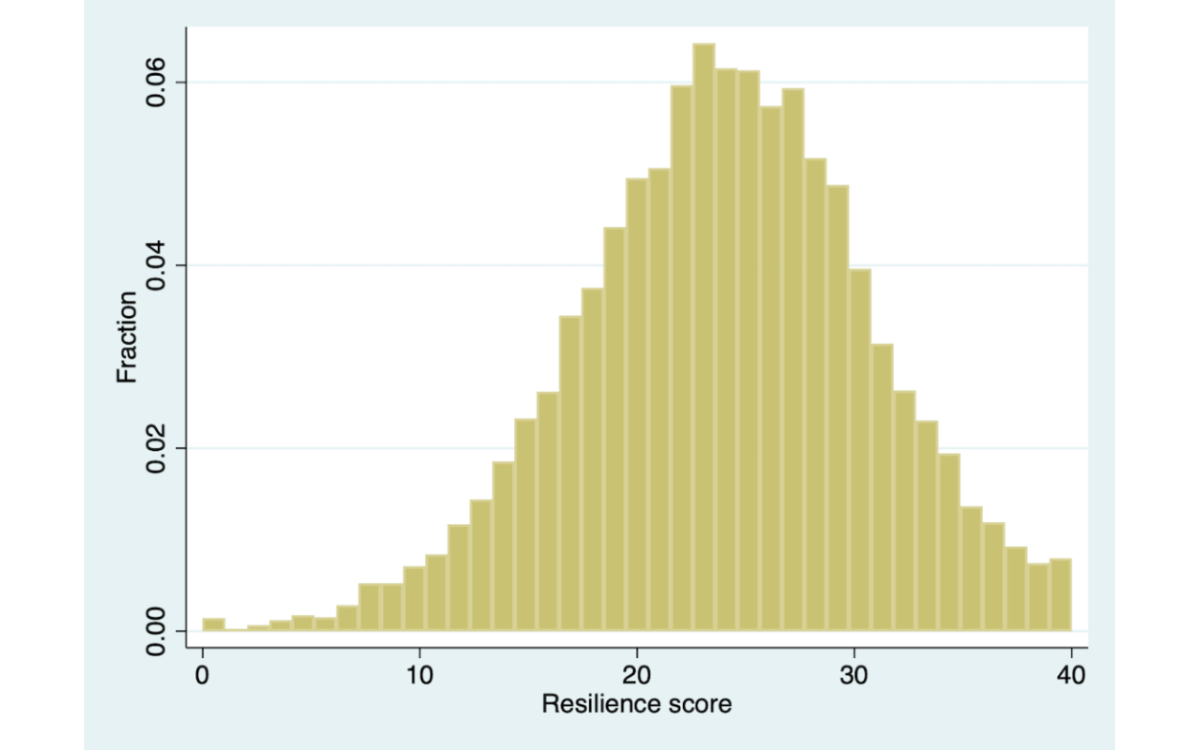
Distribution of baseline CD-RISC 10 resilience scores for 9165 members. CD-RISC 10: 10-item Connor-Davidson Resilience Scale.

Using the baseline sample of 9165 members, Figure 2 shows the distribution of baseline scores. The scores are centered on a mean of 23.84 (SD 6.56) and a median of 24 (IQR 8) out of 40, but there is significant variance in these scores. A total of 81.0% (n=7424) had a resilience score of less than 30. Table 1 shows the mean response scores across the 10 items. Responses were highest for the questions about confidence, flexibility, and perseverance, and scores were lowest for determination and weathering emotions.

Table 2 presents the number of members and baseline score statistics for the overall sample and subgroups based on demographic characteristics and mental health outcomes at baseline. Demographic data were missing for a large portion of the sample due to irregular reporting by members’ employers or health plans. Of those without missing demographic data, the majority were female and between 18 and 34 years old. Members were most likely to live in the West and South, but all four census regions were represented in the baseline sample.

For each category (gender, age, etc), a *P* value is presented in the category’s first row testing whether the difference in mean scores across the category was statistically significant. For all categories except gender and census region, the mean baseline score was statistically different across groups at the 1% level. In the row for groups with missing data, the *P* value corresponds to a *t* test of the difference in mean baseline scores between those with and without data.

For all categories, members with missing data had significantly different scores than those without missing data. The mean scores for those with missing demographic data were lower than for those who were not missing data. While we do not have direct evidence regarding why this is the case, we can at least conclude that the resilience of members with parent organizations (ie, employers or health plans) that did not send demographic data was lower than for members with parent organizations that did report these data. The mean resilience scores for members with missing PHQ-9 and GAD-7 scores followed a different pattern in that they fell between the mean scores for clinical and subclinical members. For both the PHQ-9 and the GAD-7, the mean scores for members with missing data were considerably closer to those of subclinical members, suggesting that the set of members with missing PHQ-9 and GAD-7 data were disproportionately subclinical, relative to the sample overall.

**Figure 3:**
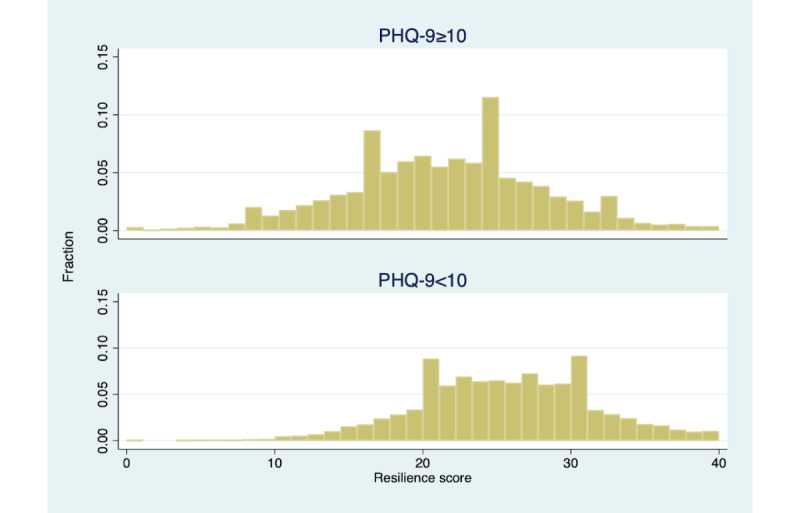
Distribution of CD-RISC 10 resilience scores by PHQ-9 threshold. CD-RISC 10: 10-item Connor-Davidson Resilience Scale; PHQ-9: 9-item Patient Health Questionnaire.

**Figure 4:**
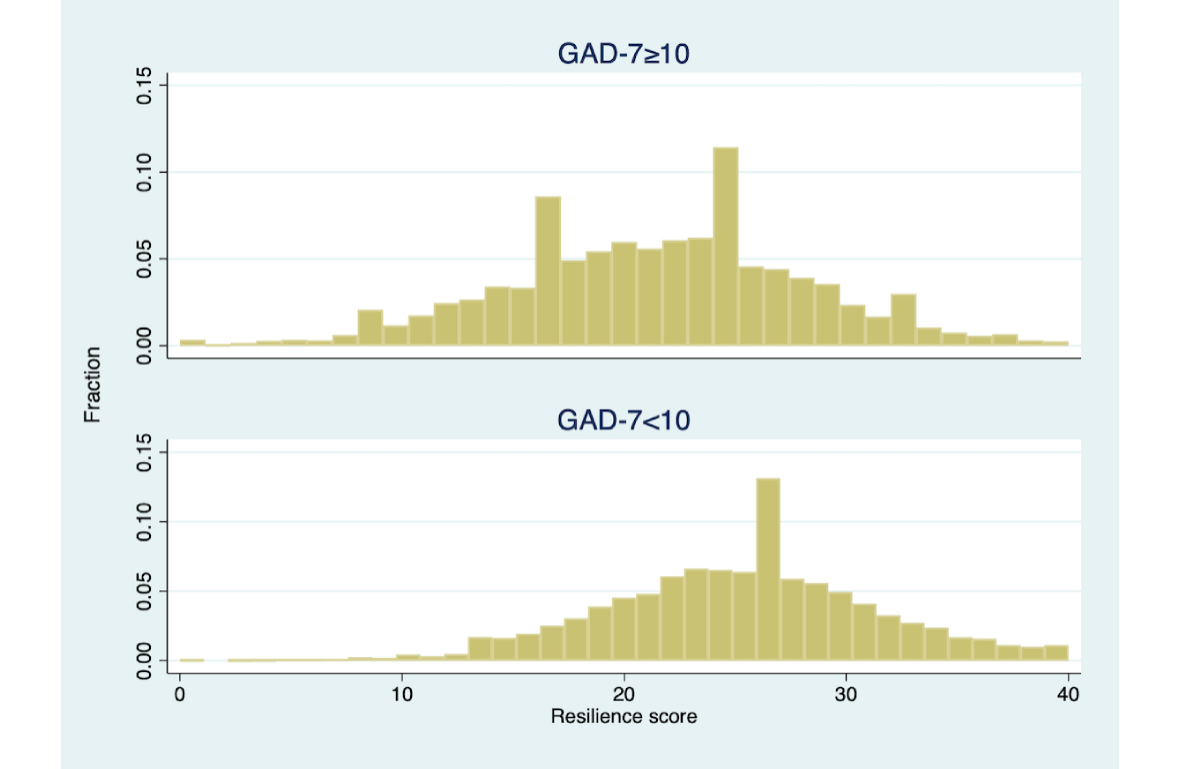
Distribution of CD-RISC 10 resilience scores by GAD-7 threshold. CD-RISC 10: 10-item Connor-Davidson Resilience Scale; GAD-7: 7-item Generalized Anxiety Disorder scale.

A significant portion of the sample screened positive for moderate to severe depression, based on the PHQ-9, or moderate to severe anxiety, based on the GAD-7. While anxiety and depression symptoms correlated with baseline scores (Table 3), there was significant overlap in the distribution of resilience scores for members with and without clinical depression or anxiety. Figures 3 and 4 display these distributions. Notably, a significant portion of members without clinical depression (3659/4841, 75.6%) and members without clinical anxiety (3907/5130, 76.2%) had low resilience.

**Table 1:** Baseline scores for individual questions.

Question topic	CD-RISC 10^a^ score, mean (SD)
Confidence	2.67 (0.95)
Determination	1.92 (1.02)
Flexibility	2.65 (0.83)
Focus	2.19 (0.99)
Grit	2.51 (1.02)
Perseverance	2.70 (0.93)
Personal growth	2.25 (0.97)
Positivity	2.36 (1.03)
Self-reliance	2.53 (0.86)
Weathering emotions	2.10 (0.99)

^a^CD-RISC 10: 10-item Connor-Davidson Resilience Scale; each item is rated on a scale from 0 (not true at all) to 4 (true nearly all the time).

**Table 2:** Baseline characteristics.

Variable		Participants (N=9165), n (%)	Mean (SD)	Median (IQR)	*P* value^a^
All participants		9165 (100)	23.84 (6.56)	24 (8)	N/A^b^
**Gender**					
	Female	3543 (38.7)	23.91 (6.54)	24 (8)	.33
	Male	1630 (17.8)	24.10 (6.83)	24 (8)	
	Missing gender	3992 (43.6)	23.66 (6.47)	24 (9)	.03
**Age (years)**					
	18-24	525 (5.7)	22.87 (6.06)	23 (8)	<.001
	25-34	2368 (25.8)	24.08 (6.24)	24 (8)	
	35-44	1233 (13.4)	24.46 (6.65)	25 (9)	
	45-64	951 (10.4)	24.21 (6.73)	25 (9)	
	≥65	48 (0.5)	23.67 (7.03)	23 (10)	
	Missing age	4040 (44.1)	23.54 (6.71)	24 (9)	<.001
**US Region**					
	West	2408 (26.3)	23.88 (6.46)	24 (8)	.39
	Midwest	762 (8.3)	24.15 (6.25)	24 (8)	
	South	2394 (26.1)	23.91 (6.83)	24 (9)	
	Northeast	1195 (13.0)	24.22 (6.31)	25 (8)	
	Missing region	2406 (26.3)	23.43 (6.61)	24 (9)	<.001
**Baseline CD-RISC 10^c^ resilience score**					
	High resilience (score ≥30)	1741 (19.0)	33.00 (2.68)	32 (4)	<.001
	Low resilience (score <30)	7424 (81.0)	21.69 (5.21)	22 (7)	
**Baseline PHQ-9^d^ score**					
	Score ≥10	3641 (39.7)	21.41 (6.67)	22 (9)	<.001
	Score <10	4841 (52.8)	25.47 (5.94)	26 (7)	
	Missing score	683 (7.5)	25.18 (6.25)	26 (8)	<.001
**Baseline GAD-7^e^ score**					
	Score ≥10	3352 (36.6)	21.43 (6.69)	22 (9)	<.001
	Score <10	5130 (56.0)	25.23 (6.04)	25 (8)	
	Missing score	683 (7.5)	25.18 (6.25)	26 (8)	<.001

^a^*P* values were based on Welch 2-tailed *t* tests, which were used to analyze differences in means across subgroups; values are reported in the first row of a category with multiple subgroups. In the row for groups with missing data, the *P* value corresponds to a *t* test of the difference in mean baseline scores between those with and without data.

^b^N/A: not applicable; *P* values were not calculated for this variable.

^c^CD-RISC 10: 10-item Connor-Davidson Resilience Scale.

^d^PHQ-9: 9-item Patient Health Questionnaire.

^e^GAD-7: 7-item Generalized Anxiety Disorder scale.

**Table 3:** Ordinary least squares regression of baseline resilience scores (N=9165).

Variable			Outcome: baseline resilience score (0-40), b (SE)^a^	*P* value
**Gender**				
	Female	Reference		N/A^b^
	Male	0.15 (0.188)		.43
	Missing gender	0.025 (0.186)		.90
**Age (years)**				
	18-24	Reference		N/A
	25-34	0.54 (0.28)		.052
	35-44	0.6 (0.311)		.053
	45-64	0.54 (0.323)		.09
	≥65	–0.39 (1.01)		.70
	Missing age	0.38 (0.269)		.16
**US region**				
	West	Reference		N/A
	Midwest	0.026 (0.254)		.92
	South	0.14 (0.185)		.46
	Northeast	0.077 (0.217)		.73
	Missing region	–0.26 (0.222)		.24
**Baseline PHQ-9^c^ score**				
	Score ≥10	Reference		N/A
	Score <10	2.3 (0.171)		<.001
**Baseline GAD-7^d^ score**				
	Score ≥10	Reference		N/A
	Score <10	2 (0.168)		<.001
**Suicidal ideation**				
	Absent	Reference		N/A
	Present	–2.1 (0.206)		<.001
Missing PHQ-9 and GAD-7 scores			3.5 (0.278)	<.001
Constant			21 (0.305)	<.001

^a^The *R*^2^ value is 0.12 and the adjusted *R*^2^ value is 0.12.

^b^N/A: not applicable; no coefficient or *P* value was calculated for the reference values.

^c^PHQ-9: 9-item Patient Health Questionnaire.

^d^GAD-7: 7-item Generalized Anxiety Disorder scale.

Table 3 presents results from a multivariate OLS regression using demographic and baseline mental health outcomes to predict baseline resilience scores. Point estimates and standard errors are included in the table. Holding all else constant, gender does not significantly predict scores. The youngest members (aged 18 to 24 years) had significantly lower scores than those aged 25 to 64 years and higher scores than those aged 65 years and above; the latter difference was not statistically significant. There were no statistically significant differences across census regions. Baseline scores for members with no or mild depression or anxiety were 2.3 or 2.0 points higher, respectively, than those with moderate to severe depression or anxiety. Consistent with the hypothesis that members with missing PHQ-9 and GAD-7 scores are more similar to subclinical members, the scores for these members were 4.3 points higher than for members with clinical depression (ie, the leave-out comparison group). Self-reported suicidal ideation was associated with a significant reduction of 2.1 points in baseline resilience.

## Discussion

### Principal Findings

This study found that individuals accessing the Ginger system had, on average, low baseline resilience levels (median CD-RISC 10 score of 24), well below prior benchmarks of the US general population and in line with studies of veterans with PTSD and depression [20]. This is not surprising given that the sample includes individuals seeking out mental health services and the data collection period, which coincided with spikes of COVID-19 cases and other disruptive world events. A significant portion of the sample screened positive at baseline for moderate to severe depression (3641/8482, 42.9%, based on the PHQ-9) and moderate to severe anxiety (3352/8482, 39.5%, based on the GAD-7). A total of 81.0% of members had a baseline resilience score of less than 30.

Our descriptive analysis shows that younger members tended to have lower resilience, which is consistent with several studies of adolescents and young adults in the United States [3,21]. Baseline resilience scores for members with no or mild depression or anxiety were higher than those for members with moderate to severe depression or anxiety. This association was even stronger for members who reported suicidal ideation. This is consistent with findings from other studies showing that self-reported mental health diagnoses were negatively associated with higher resilience, and adults with reported low or normal levels of resilience were more likely to experience mental distress compared to those with high resilience [11,21]. However, it is interesting to note that despite having relatively higher resilience scores, members with no or mild depression or anxiety still had low resilience scores on average. This highlights the need for mental health support among individuals who might not typically be recommended for treatment based on traditional clinical assessments, like the PHQ-9 and the GAD-7.

### Strengths and Limitations

A major strength of this study was the large number of participants; this is one of the largest studies using the CD-RISC 10 measure, which allows for certain subgroup analyses. This is also one of the first applications of this measure in a large-scale real-world setting, in contrast to smaller controlled research settings. Incorporating a strength-based measure like resilience, in contrast to symptom-based measures like the PHQ-9 and the GAD-7, allows us to better understand the needs of individuals seeking mental health services.

There are several limitations to this study. Of the 9165 Ginger participants in the study, 43.6% had missing data for gender, and 44.1% had missing data for age. Typically, these demographic data are shared by members’ organizations through which they access Ginger services (eg, employers). Some organizations do not share demographic data. Given the amount of missing data in our sample, we acknowledge the need for further research that focuses on the relationship between resilience and demographics. In part due to incomplete data reporting by parent organizations, Ginger has launched the capability for members to elect to self-report their demographic information in the app. These self-reported data will supplement the parent organization–reported data and will be available for future research projects. This functionality had not been launched by the time of this analysis. For the purposes of this study, we presented results for members with missing data and we controlled for whether a member’s demographic data were missing in any regression analyses. However, because the Ginger platform is offered through employers, the survey respondents were working-age adults, which suggests that these findings may generalize to the professional workforce and those enrolled in health benefits through their employer. Further, the current data did not include information on other sociodemographic or contextual factors (eg, marital status, family composition, significant life events, sources of social support, and educational level) that might be related to resilience and mental health, and that may have been of particular significance during the pandemic.

### Future Research

Given that behavioral health providers often focus on clinical symptoms, such as those measured by the PHQ-9 and the GAD-7, a deeper understanding of nonclinical outcomes, such as resilience, is increasingly important to the growing digital behavioral health industry. This is especially true given that many members seeking behavioral health support do not experience clinical symptoms. For example, the majority of members in this study screened negative for depression and anxiety, signaling a need to not only track other outcomes that are associated with members’ well-being but also to understand which specific interventions have an impact on these outcomes, the expected size of these impacts, and which subpopulations may respond differentially. This study points to many directions for future research, primarily looking at how these scores evolve over time, with a particular focus on whether the interaction of resilience and clinical symptoms (eg, depression and anxiety) impacts members’ responses to behavioral health interventions. Given that this data set included PHQ-9 and GAD-7 scores, future studies could examine the relationship between resilience and symptoms of anxiety and depression, for example, whether increased baseline resilience is associated with a higher likelihood of symptom improvement and faster time to improvement (ie, less use of care services). Additionally, future research could look at more detailed classifications of use and conversational features extracted via natural language processing of text messages to better understand which factors have stronger associations with increased resilience.

### Conclusions

Resilience is a construct often referred to but less often defined and measured, particularly in clinical settings that tend to focus more on symptom-based measures. In this study, we found that members accessing mental health services from January to August 2021 had extremely low baseline resilience, in line with prior studies of trauma survivors, which highlights the need for expanding access to care. Overall, younger members and those with higher levels of depression and anxiety at intake reported lower levels of resilience at baseline. Notably, members with no or mild depression or anxiety still had low resilience scores on average, demonstrating the need for mental health support among individuals without clinical symptoms. Future research will examine changes in resilience over time in addition to factors associated with those changes.

## Abbreviations

CD-RISC: Connor-Davidson Resilience Scale
CD-RISC 10: 10-item Connor-Davidson Resilience Scale
GAD-7: 7-item Generalized Anxiety Disorder scale
IRB: Institutional Review Board
OLS: ordinary least squares
PHQ-9: 9-item Patient Health Questionnaire
PTSD: posttraumatic stress disorder
